# Impact of potential modifications to Alzheimer’s disease clinical trials in response to disruption by COVID-19: a simulation study

**DOI:** 10.1186/s13195-021-00938-w

**Published:** 2021-12-20

**Authors:** Lon S. Schneider, Yuqi Qiu, Ronald G. Thomas, Carol Evans, Diane M. Jacobs, Shelia Jin, Jeffrey A. Kaye, Andrea Z. LaCroix, Karen Messer, David P. Salmon, Mary Sano, Kimberly Schafer, Howard H. Feldman

**Affiliations:** 1 Keck School of Medicine of the University of Southern California, Los Angeles, USA; 2 University of California, San Diego, USA; 3 Oregon Health Sciences University, Portland, USA; 4 Icahn School of Medicine at Mount Sinai, New York, USA

**Keywords:** COVID-19, Alzheimer, Clinical trials, Simulations, Mild cognitive impairment, Disease modification, Symptomatic treatment

## Abstract

**Background:**

The COVID-19 pandemic disrupted Alzheimer disease randomized clinical trials (RCTs), forcing investigators to make changes in the conduct of such trials while endeavoring to maintain their validity. Changing ongoing RCTs carries risks for biases and threats to validity. To understand the impact of exigent modifications due to COVID-19, we examined several scenarios in symptomatic and disease modification trials that could be made.

**Methods:**

We identified both symptomatic and disease modification Alzheimer disease RCTs as exemplars of those that would be affected by the pandemic and considered the types of changes that sponsors could make to each. We modeled three scenarios for each of the types of trials using existing datasets, adjusting enrollment, follow-ups, and dropouts to examine the potential effects COVID-19-related changes. Simulations were performed that accounted for completion and dropout patterns using linear mixed effects models, modeling time as continuous and categorical. The statistical power of the scenarios was determined.

**Results:**

Truncating both symptomatic and disease modification trials led to underpowered trials. By contrast, adapting the trials by extending the treatment period, temporarily stopping treatment, delaying outcomes assessments, and performing remote assessment allowed for increased statistical power nearly to the level originally planned.

**Discussion:**

These analyses support the idea that disrupted trials under common scenarios are better continued and extended even in the face of dropouts, treatment disruptions, missing outcomes, and other exigencies and that adaptations can be made that maintain the trials’ validity. We suggest some adaptive methods to do this noting that some changes become under-powered to detect the original effect sizes and expected outcomes. These analyses provide insight to better plan trials that are resilient to unexpected changes to the medical, social, and political milieu.

## Background

The unexpected COVID-19 pandemic substantially disrupted ongoing randomized clinical trials for Alzheimer disease (AD), including seven trials being conducted by the Alzheimer’s Disease Cooperative Study (ADCS). In the first months of the pandemic, California and many other states instituted population-wide “stay-at-home” orders to protect vulnerable individuals and to slow the spread of the virus. These orders prevented planned in-person study visits, forcing investigators to make decisions to prematurely end trials, pause trials in place, or change trial methods with adaptations such as extending treatment, delaying outcomes, or adopting remote assessment procedures. Investigators and sponsors were challenged to make these changes while maintaining trial validity and clinical meaningfulness by mitigating the risks of introducing bias or loss of statistical power to detect a potential therapeutic effect. Unfortunately, little was known about the impact of many of these modifications on the integrity of typical AD randomized, controlled trials to guide trial decisions.

To better understand the effects of exigent trial modifications due to disruptions caused by the COVID-19 pandemic, we developed several scenarios with different potential trial modifications and simulated the impact of making these modifications on the power to detect a specified treatment effect. Simulations and statistical modeling were conducted with data from past therapeutic trials conducted by the ADCS and adjusted for patterns of enrollment, follow-up, and dropouts that occurred in those trials. Our goal was to show the potential effects of various trial modifications that might have been taken in reaction to disruptions caused by COVID-19 to inform both current trials and future trial planning.

## Methods

We created two trial constructs based on common characteristics of the AD randomized controlled trials, one for a symptomatic treatment and one for a disease modification treatment. Each construct was modeled after a similar symptomatic or disease modification treatment trial that had been conducted by the ADCS. Within both trial constructs, we assumed that in-person visits were paused for a 6-month period from the date of a strict stay-at-home order until the order was relaxed, and in-person visits could resume. Within each construct, we assessed three representative hypothetical scenarios that differed in modifications made in response to the abrupt interruption of in-person visits. Simulations of the three scenarios were performed using data from past ADCS trials to investigate the impact of trial modifications on the power to detect a specified treatment effect. The details of the trial constructs, including trial status at the time the stay-at-home order was issued, protocol modifications due to the stay-at-home order, and specific modeling assumptions and procedures, are described and summarized in Table 1. Assumptions regarding COVID-related trial modifications differed in the two trial constructs; therefore, different approaches and methods were used in simulations. All simulations were conducted in R version 3.6.3.

**Table 1:** Three representative COVID-19 scenarios for a symptomatic and a disease modification trial. No in-clinic visits are allowed during a 6-month interruption from March 19 to September 19, 2020. Bold text indicates significant distinctions between the two trials

	Symptomatic trial Phase 2 **symptomatic trial** in mild-to-moderate AD • **Daily oral** medication • Planned outcomes at **12 months** • *N* = 360 randomized to drug or placebo	Disease modification trial Phase 2/3 **disease modification trial** for early AD • In-clinic **monthly drug infusions** • Planned outcomes at **18 months** • *N* = 280 randomized to drug or placebo
Trial status on March 19, 2020	Fully enrolled	Fully enrolled
	97.5% completed 3 months	80% completed 3 months
	67% completed 6 months	50% completed 6 months
	45% completed 9 months	25% completed 12 months
	22% completed 12 months	12% completed 18 months
Dropout	30% dropout rate evenly distributed across visits. No discontinuation due to COVID19	24% (66 subjects) discontinued before or on March 19 (partially due to COVID19). An additional non-COVID dropout rate of 25%. Final dropout rate: 42%^a^
Scenario 1	Trial stopped on March 19; no further visits or data collection	
Scenario 2	Outcome assessments paused during COVID interruption, resumed after interruption.	
	• ~ 50%, who had completed 9 months, missed their 12-month outcome • ~ 25% missed their 9-month outcome but could have 12-month assessments • ~ 30% missed other outcomes **Medication continued** during COVID interruption.	• ~ 50%, who had completed 12 months, missed their 18-month outcome • ~ 25% missed their 12-month outcome but could have 18-month assessments • ~ 50% missed other outcomes **Medication infusion paused** during COVID interruption, resumed after interruption.
Scenario 3	**No outcomes assessed** during COVID interruption. **Medication continued** during COVID, with extended medication provided beyond 12 months **Up to a 3-month extension of the final assessment window**, to allow completion of 12 months outcomes after clinics reopened	**Outcomes assessed remotely** during COVID interruption; in-clinic assessment resumed after interruption. **Medication infusion paused** during COVID interruption **No extension of the final assessment window**

^a^The original expected dropout rate is 25%. In this scenario, 66 discontinued due to COVID-19, and because some would have been “potential completers,” the final dropout rate is approximately 42%. See trial construct 2 statistical methods

### Trial construct 1—Symptomatic trial

The first hypothetical trial construct is conceptualized as a phase 2 proof-of-concept, 12-month, randomized, double-blind, placebo-controlled trial of an oral agent for mild-to-moderate AD with change on the Alzheimer’s Disease Assessment Scale-Cognitive (ADAS-cog) as the primary outcome measure. Assessments were planned at baseline, 3 months, 6 months, and 12 months (endpoint). The trial was powered with 0.80 statistical power (*α* = 0.05) to detect a 2.6-point ADAS-cog difference between baseline and 12-month (endpoint) scores in a two-sided, two-sample *t* test analysis with 180 individuals required per arm (drug or placebo). We assumed that at the time of the pause, the trial was fully enrolled: a total of 360 individuals had been randomized to drug or placebo and had completed a baseline evaluation; 97.5% of participants had completed 3-month follow-up; 67% had completed 6 months; 45% had completed 9 months; and 22% had completed the planned 12-month endpoint. We modeled three potential scenarios in response to the pandemic stay-at-home order.

#### Scenario 0 (“trial as planned”)

We included for reference the “trial as planned” scenario that assumed no interruption to in-person assessments to confirm that original power was near 80%.

#### Scenario 1

The trial was abruptly ended on the date of the stay-at-home order, and no further outcome data were collected. In this base condition, the hypothetical trial was truncated and data available as of the date of the stay-at-home order were analyzed.

#### Scenario 2

Trial medications continued throughout the pause; outcome assessments were stopped for 6 months and then resumed after the pause; windows for outcome assessments were not extended, so assessments that were due during the pause were missing. Because there was no extension for visits to occur outside of the 12-month trial window, the 45% of individuals who had completed through month 9 before the pause did not have a chance for another assessment within the 12-month window, the 31.5% of individuals who completed only through month 6 before the pause did not have a month 9 assessment but did have a month 12 (endpoint) assessment, and the 2.5% of individuals who completed only through month 3 did not have a month 6 assessment but did have month 9 and month 12 (endpoint) assessments.

#### Scenario 3

Trial medications continued throughout the pause; outcome assessments were stopped for 6 months and then resumed after the pause; the window to complete the 12-month endpoint assessment was extended by 3 months so that endpoint outcomes could be assessed in person. Extending the window for the 12-month endpoint assessment represented a protocol change that allowed patients to be on study drug for up to 15 months. As a result, 22% of individuals would have been treated for the planned 12 months prior to the pandemic (i.e., completed before the pause); the remainder of study participants could have been treated for a longer period of up to 15 months.

#### Statistical methods for trial construct 1

For each scenario, we performed a simulation to calculate power to detect each of three different 12-month ADAS-Cog change score effect sizes for placebo vs. treatment (2.0, 2.5, and 3.0 ADAS-cog points, pooled standard deviation (SD) fixed at 6.0) using three different analysis methods (linear mixed effects with categorical time, linear mixed effects with continuous time, and Student’s *t* test) in three different sample sizes (the planned sample size of 360 and two others: 320, 400). Simulations were done with Monte Carlo methods applied to a pooled dataset of ADAS-cog scores from 641 participants who completed the ADCS homocysteine and lipid-lowering trials [1, 2].

**Fig. 1:**
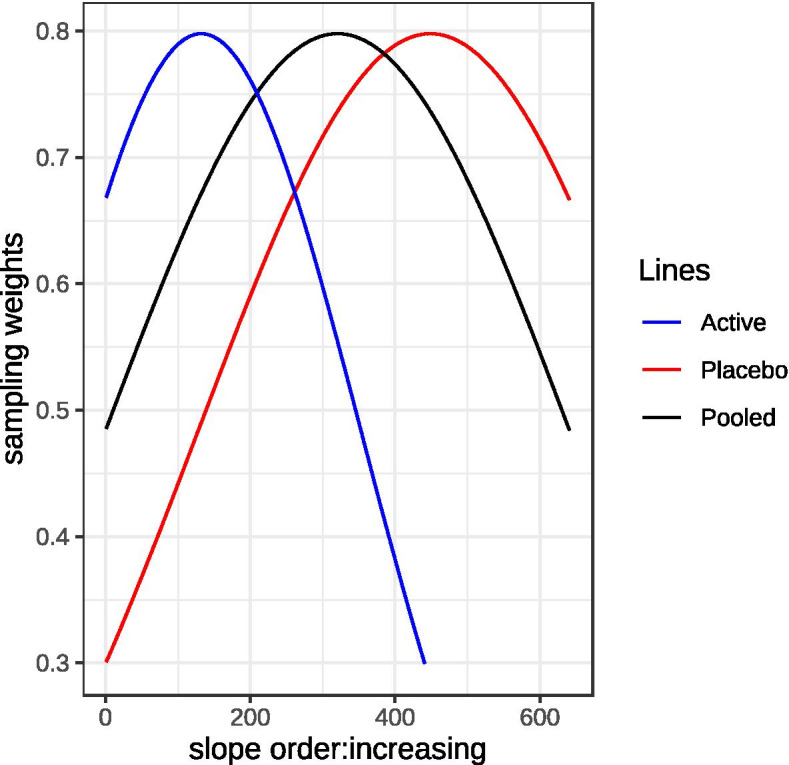
Weights distributions for placebo (red) and active group (blue) sample selection from a set of 641 ordered slopes

As a first step, a least squares slope statistic was computed for the ADAS-Cog scores of each participant in the pooled dataset as a measure of relative disease progression over a 12-month study period. This set of slopes was ordered from smallest to largest. The following algorithm was then applied. (1) An N/2 sized subset of these slopes was selected at random without replacement using sampling weights biased toward larger slopes. These slopes represented the “placebo group.” (2) A second N/2 sized subset of slopes was selected in a similar manner but using sampling weights biased toward smaller slopes. These slopes represented the “active drug group.” The weight distributions for “placebo group” and “active drug group” sample selection are shown in Fig. 1. (3) The sampling procedure was repeated to allow subsets of slopes to be drawn with means and SDs for ADAS-cog change differences (i.e., active vs. placebo effect size) centered around the targeted ADAS-cog change difference score (i.e., 2.0, 2.5, or 3.0 ADAS-cog points) and SD (i.e., 6.0 SD), and this repetition continued until the observed mean ADAS-cog change difference score and SD were within 0.001 of the targeted effect size and SD. This method of creating a dataset was repeated until we obtained 20 datasets of size *N* with the specified observed between-group ADAS-Cog change difference (i.e., 2.0, 2.6 or 3.0) and SD (i.e., 6.0).

As a second step, a filtering process was applied to each dataset such that 30% of subjects were randomly selected to be dropped from the study prior to the 12-month endpoint. The timing of the dropouts was uniformly distributed over the 12-month trial period. Following the application of the dropout filter, one of three additional filters was applied to represent one of the COVID response modification scenarios (plus the “as-planned” scenario) described above. The 20 datasets for each combination of sample size, effect size, and scenario were submitted to a bootstrap analysis with 1000 replicates to determine the statistical power of each of three statistical testing procedures: (a) linear mixed effects with categorical time, (b) linear mixed effects with continuous time, and (c) Student’s *t* test. The bootstrap process involved resampling the dataset multiple times without replacement and calculating the three test statistics and their associated *P* values. The proportion of *P* values less than 0.05 over the 1000 replicates provided the power estimate.

### Trial construct 2—Disease modification trial

The second hypothetical trial construct is conceptualized as a phase 2b or 3 disease modification trial with monthly intravenous infusions of a monoclonal antibody targeted to AD pathology. It is an 18-month, randomized, double-blind, placebo-controlled trial for early-stage AD (i.e., MCI due to AD, mild AD dementia) with the ADAS-cog as the primary outcome. Evaluations were planned for baseline, 3 months, 6 months, 12 months, and 18 months (endpoint). The trial was power with 0.80 statistical power (*α* = 0.05) to detect a 1.85-point ADAS-cog difference between treatments at 18 months using a two-sided, two-sample *t* test analysis with 140 individuals per arm (drug or placebo). We assumed that at the time of the pause, the trial was fully enrolled: a total of 280 individuals had been randomized to drug or placebo and had completed a baseline evaluation, 80% had completed 3-month follow-up, 50% had completed 6 months, 25% had completed 12 months, and 12% had completed the planned 18-month endpoint. We modeled three potential scenarios in response to the pandemic stay-at-home order.

#### Scenario 0 (“as planned”)

We included for reference the “as planned” scenario that assumed no interruption to in-person assessments to confirm that original power was near 80%.

#### Scenario 1

The trial was abruptly ended on the date of the stay-at-home-order, and no further outcome data were collected. In this base condition, the hypothetical trial was truncated and data available as of the date of the stay-at-home order were analyzed.

#### Scenario 2

Medication and assessments were stopped for 6 months and then resumed after the pause. This resulted in missed medication and assessments for visits planned during the pause creating a condition where approximately 20% of individuals who only completed baseline missed their month 3 and 6 outcome assessment, approximately 30% of individuals who had completed month 3 missed their month 6 outcome assessment, approximately 25% who completed month 6 had missed month 12, and approximately 13% who completed month 12 missed month 18. We also assumed that 24% discontinued before or on the date of the pause.

#### Scenario 3

Trial medication was stopped during the 6-month pause, but the cognitive outcome measure continued to be assessed remotely on the planned schedule. The impact of remote assessment was modeled by adjusting ADAS-Cog scores by an increase (worsening) of 0.5 points. Medication was resumed after the pause. This created a condition similar to scenario 2’s scheme, but they had remote assessments rather than missing the assessments. We again assumed that 24% discontinued before or on the date of the pause.

#### Statistical methods for trial construct 2

For each scenario, we performed a simulation to calculate power to detect each of four different ADAS-Cog effect sizes for placebo vs. treatment (1.5, 2.0, 2.5, and 3.0 ADAS-cog points, pooled SD fixed at 4.7) and the well-powered effect size 1.85 using three different analysis methods (linear mixed effects with categorical time, linear mixed effects with continuous time, and Student’s *t* test) in three different sample sizes (the planned sample size of 280 and two others: 240, 320). Simulations were based on resampling data from 769 participants with MCI from the ADCS donepezil vs. vitamin E trial [3], a trial with inclusion criteria and an assessment schedule (i.e., 3, 6, 12, and 18 months) similar to trial construct 2. Accrual and dropout patterns from this trial informed the simulation studies.

Statistical power under each of the three scenarios and various effects and sample sizes was determined by simulations, with each simulation size *N* = 1000. Using the planned 280 participant sample size as an example, each simulation run resampled 280 participants from the donepezil vs. vitamin E trial population. We used stratified sampling with replacement to retain the dropout pattern in the donepezil vs. vitamin E trial, drawing 75% of each sample from completers (the proportion of completers in the donepezil vs. vitamin E trial) and 7.5% from those who dropped before 18 months (the proportion of dropouts before 18 months in the donepezil vs. vitamin E trial). Sampled (and resampled) participants were randomly assigned 1:1 to the “active drug” or the “placebo” arm. Participants were then randomly assigned to one of four predetermined accrual/start dates in the proportions described for trial construct 2, so that at the time of the stay-at-home order, 80% had completed 3 months, 50% had completed 6 months, 25% had completed 12 months, and 12% had completed the planned 18-month endpoint. After this assignment, 24% were randomly selected to have “dropped out” on the day of the stay-at-home order (i.e., ADAS-Cog data beyond their presumed last assessment before the stay-at-home order were deleted). In this way, we simulated a 24% dropout due to the pandemic, overlaid on the naturally occurring dropout in the trial.

The treatment effect was constructed as follows: using the observed placebo arm, we first established that a mean difference of 1.85 points between arms in 18-month ADAS-Cog change scores (a standardized effect size of 0.395) would be detectable at 80% power with a sample size of 280 and 24% drop out rate, using a two-sided *t* test at 95% significance level. We centered our simulated effect sizes around this value for the power studies, which were carried out for the mean between-arm differences in 18-month ADAS-Cog change scores of 1.5, 2.0, 2.5, and 3.0 points. For all scenarios, the effect of active treatment was modeled by adding a quadratic time trend (i.e., an increasing treatment effect) to the outcome data for the “active drug” arm in each simulation.

Each simulated dataset was used to determine power to detect a significant difference at the 5% level (two-sided) between “active drug” and “placebo” arms using three methods: (a) a *t* test comparing the difference at 18 months; (b) a linear mixed effects model with categorical time, testing for a difference at 18 months; and (c) a linear mixed effects model with continuous time, testing for a difference in slopes. Models included fixed effects of baseline ADAS-Cog score, treatment group, and baseline ADAS-Cog score x treatment group interaction. Assessment visit (e.g., 3 months, 6 months) and the assessment visit x treatment group interaction were also included. The categorical time model assumed a random intercept. The continuous time model assumed an unstructured covariance matrix.

The programming code for both trial constructs are available on GitHub at https://github.com/ucsandiego-adcs/manuscript-adcs-covid-19-white-paper.

## Results

### Trial construct 1—Symptomatic trial

**Fig. 2:**
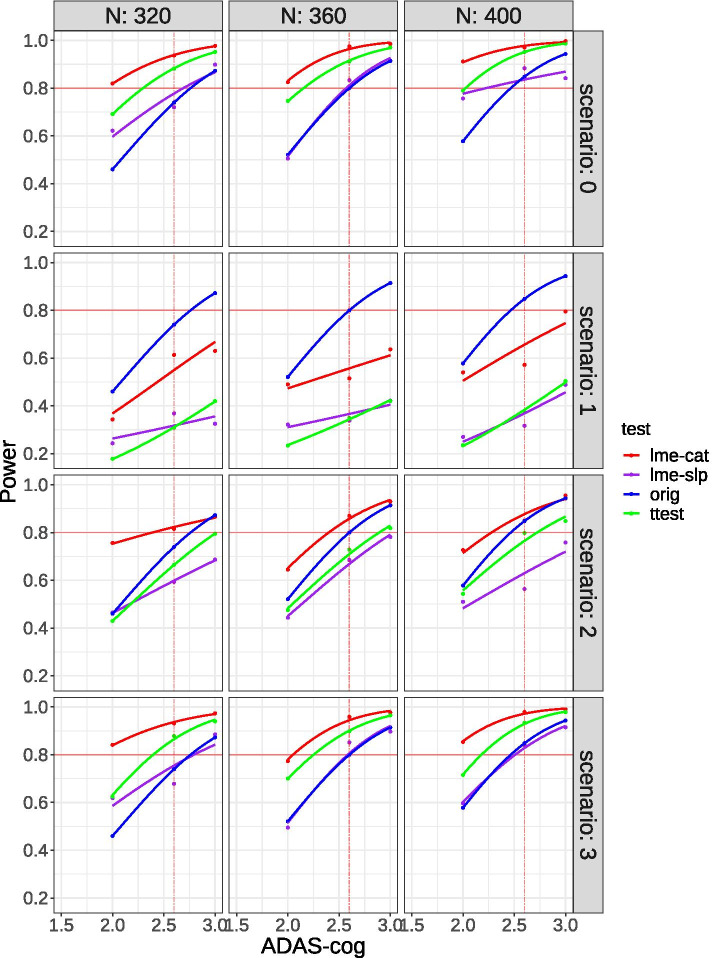
Simulation results by sample size and scenario for symptomatic trials. There are two MMRM tests per facet: categorical time model (lme-cat), red, and continuous time model (lme-slp), purple. The original *t* test (*z* test), blue, power calculation is added to each plot. The green (*t* test) curve represents the simulation results evaluated using a simple *t* test. The data points were smoothed using the stat smooth command in the ggplot2 R package, with the link function set to probit

The impact of each trial modification scenario on the power to detect 12-month ADAS-Cog change effect sizes (placebo vs. treatment) of 2.0, 2.5, or 3.0 ADAS-cog points under each of four different statistical analysis approaches are shown for three different sample sizes (*n* = the planned 360, 320, or 400 participants) in Fig. 2. To illustrate how various trial modification scenarios and analysis approaches affected power in the planned trial, a red horizontal solid line indicating 80% power and a red vertical dashed line indicating the expected 2.6-point difference in ADAS-cog change between arms (placebo vs. treatment) are shown on each set of smoothed power curves. Each statistical analysis approach is plotted separately (in different colors). For reference, the power curve for the *t* test analysis under the original trial conditions (i.e., no impact of COVID-19 is assumed) is also shown.

Focusing on the results for the planned sample size of 360 (Fig. 2, middle column), the trial as originally planned (scenario 0, no impact of COVID) was powered at or above the stated 80% to detect the expected 2.6-point difference in ADAS-cog change regardless of the statistical analysis approach used. Power to detect the expected difference was higher for the linear mixed effects with categorical time and Student’s *t* test approaches (approximately 90% or higher) than for the linear mixed effects with continuous time approach. The impact of trial modifications made under each scenario is presented below.

#### Scenario 1 (the trial is stopped)

With the planned sample size of 360 (Fig. 2, middle column), power to detect the expected 2.6-point difference in ADAS-cog change was greatly reduced and fell below 0.60 regardless of the statistical analysis approach applied. The linear mixed effects with categorical time approach provided more power (0.51) than the linear mixed effects with continuous time (0.35) or *t* test (0.33) approaches. If the expected effect size was increased to a 3.0-point difference in ADAS-cog change, and/or sample size was increased to 400 (Fig. 2, right column), power remained below 80% regardless of the statistical analysis approach used.

#### Scenario 2 (medication continued; assessments paused but resumed after 6 months; fixed 12-month endpoint)

With the planned sample size of 360 (Fig. 2, middle column), power to detect the expected 2.6-point difference in ADAS-cog change using the linear mixed effects with categorical time analysis approach was 0.89, a value greater than the originally planned 0.80. In contrast, the power to detect this effect dropped below 0.80 for the linear mixed effects with continuous time (0.69) and Student’s *t* test (0.72) analysis approaches. If the expected effect size was increased to a 3.0-point difference in ADAS-cog change, power increased (as expected) for all analysis approaches and was now at or near 0.80 for both the linear mixed effects with continuous time and Student’s *t* test approaches. If the sample size was increased to 400 (Fig. 2, right column), neither the linear mixed effects with continuous time nor Student’s *t* test approaches had 0.80 power to detect the expected 2.6-point difference in ADAS-cog change. If the sample size was reduced to 320 (Fig. 2, left column), power to detect the expected 2.6-point difference in ADAS-cog change remained above 0.80 for the linear mixed effects with categorical time analysis approach (but not other approaches).

#### Scenario 3 (medication continued; assessments paused but resumed after 6 months; endpoint extended to 15 months)

With the planned sample size of 360 (Fig. 2, middle column), power to detect the expected 2.6-point difference in ADAS-cog change was 0.80 or higher regardless of the statistical analysis approach applied. The linear mixed effects with categorical time approach (0.98) and Student’s *t* test approach (0.90) had higher power than the linear mixed effects with continuous time (0.85) approach. As expected, power to detect the expected 2.6-point difference in ADAS-cog change increased for all statistical analysis approaches if the sample size was increased to 400 (Fig. 2, right column). If the sample size was reduced to 320 (Fig. 2, left column), power for the linear mixed effects with continuous time analysis approach (but not other approaches) to detect the expected 2.6-point difference in ADAS-cog change fell below 0.80 (and was below 0.80 for the original trial conditions).

As a summary, the power to detect the expected 2.6-point difference in ADAS-cog change with the planned sample size of 360 is compared across trial-modification scenarios and statistical analysis approaches in Table 2. Acceptable power (i.e., ≥ 0.80) cannot be maintained if the trial is abruptly stopped and no additional outcome data are collected (scenario 1), regardless of the analysis approach taken. However, acceptable power can be maintained with a 6-month pause in data collection (scenario 2) if the analysis approach is changed to a linear mixed effects model with categorical time. Well above acceptable power (i.e., ≥ 0.80) can be maintained with a 6-month pause in data collection, if data collection is resumed after the pause, and the trial endpoint is extended (scenario 3). This is particularly true if a linear mixed effects model with categorical time or Student’s *t* test analysis approach is applied.

**Table 2:** Trial construct 1—symptomatic trial. Power to detect an expected 2.60-point difference in ADAS-cog change in the planned sample (*n* = 360)

Trial modification	Linear mixed effects model: categorical time	Linear mixed effects model: continuous time	Student’s *t* test	Original *t* test (for reference)
**Scenario 1**	.51	.35	.33	.80
**Scenario 2**	.89	.69	.72	.80
**Scenario 3**	.98	.85	.90	.80

### Trial construct 2—Disease modification trial

**Fig. 3:**
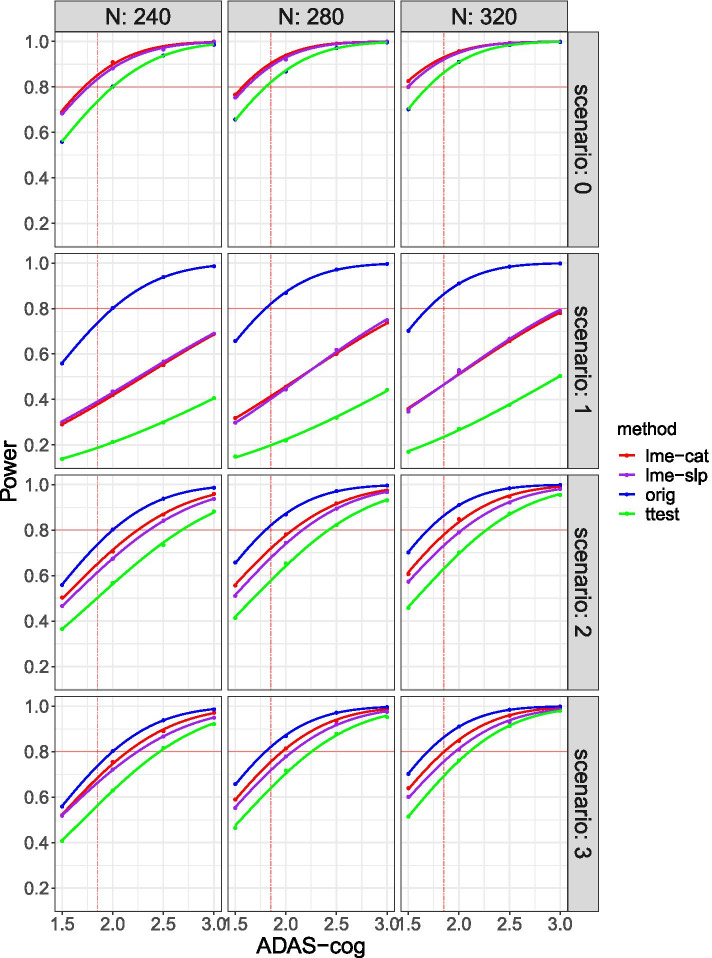
Simulation results by sample size and scenario for disease modification trials. There are three scenarios for three different approaches. The horizontal solid line (red) shows 0.80 power, and the vertical dashed line (red) indicates the 1.85-point difference in the ADAS-cog between treatment groups that the trial was originally powered to detect. The categorical time model (lme-cat, red), continuous time model (lme-slp, purple), and *t* tests (green) are presented. The blue curve shows the power of the *t* test under the original conditions with no impact of COVID-19 assumed

The impact of each trial modification scenario on the power to detect 18-month ADAS-Cog change effect sizes (placebo vs. treatment) of 1.5, 2.0, 2.5, or 3.0 ADAS-cog points under each of four different statistical analysis approaches is shown for three different sample sizes (*n* = the planned 280, 240, or 320 participants) in Fig. 3. To illustrate how various trial modification scenarios and analysis approaches affected power in the planned trial, a red horizontal solid line indicating 80% power and a red vertical dashed line indicating the expected 1.85-point difference in ADAS-cog change between arms (placebo vs. treatment) are shown on each set of smoothed power curves. Each statistical analysis approach is plotted separately (in different colors). For reference, the power curve for the *t* test analysis under the original trial conditions (i.e., no impact of COVID-19 is assumed) is also shown.

Focusing on the results for the planned sample size of 280 (Fig. 3, middle column), the trial as originally planned (scenario 0, no impact of COVID) was powered at or above the stated 80% to detect the expected 1.85-point difference in ADAS-cog change regardless of the statistical analysis approach used. As anticipated, power to detect the expected 1.85-point difference in ADAS-cog change increased for all statistical analysis approaches if the sample size was increased to 320 (Fig. 3, right column) and decreased if the sample size was decreased to 240 (Fig. 3, left column), although with *n* = 240, Student’s *t* test analysis approach fell below 0.80 power to detect the expected 1.85-point difference in ADAS-cog change. Regardless of the sample size or analysis approach, power approached 100% when the effect size was increased from a 1.85-point to a 2.5-point or higher difference in ADAS-cog change. The impact of trial modifications made under each scenario is presented below.

#### Scenario 1 (the trial is stopped)

With the planned sample size of 280 (Fig. 3, middle column), power to detect the expected 1.85-point difference in ADAS-cog change was greatly reduced and fell below 0.50 regardless of the statistical analysis approach applied. The linear mixed effects with categorical time (0.42) and linear mixed effects with continuous time (0.40) approaches were similarly powered, and both were better powered than Student’s *t* test (0.19) approach. If the expected effect size was increased to a 3.0-point difference in ADAS-cog change, and/or sample size was increased to 320 (Fig. 3, right column), power remained below 0.80 regardless of the statistical analysis approach used (although it approached 0.80 for the two linear regression analysis methods).

#### Scenario 2 (medication and assessments paused)

With the planned sample size of 280 (Fig. 3, middle column), power to detect the expected 1.85-point difference in ADAS-cog change was below 0.80 regardless of the statistical analysis approach applied. The linear mixed effects with categorical time (0.71) and linear mixed effects with continuous time (0.67) approaches were similarly powered, and both were better powered than Student’s *t* test (0.56) approach. If the expected effect size was increased to a 2.0-point difference in ADAS-cog change, power remained below 0.80 regardless of the statistical analysis approach used. However, power to detect a 2.5-point difference in ADAS-cog change was above 0.80 for all analysis methods. If the sample size was increased to 320 (Fig. 3, right column), power to detect the expected 1.85-point difference in ADAS-cog change remained below 0.80 regardless of the statistical analysis approach applied, although power approached 0.80 for the linear mixed effects with categorical time analysis approach.

#### Scenario 3 (medication paused; assessments continued remotely)

With the planned sample size of 280 (Fig. 3, middle column), power to detect the expected 1.85-point difference in ADAS-cog change was below 0.80, regardless of the statistical analysis approach applied; however, power approached 0.80 for the linear mixed effects with categorical time (0.75) and linear mixed effects with continuous time (0.71) approaches. Both were better powered than Student’s *t* test (0.64) analysis approach. If the expected effect size was increased to a 2.0-point difference in ADAS-cog change, power for the linear mixed effects with categorical time analysis (0.81), but not the other analysis approaches, was greater than 0.80. Power to detect a 2.5-point difference in ADAS-cog change was above 0.80 for all analysis methods. If the sample size was increased to 320 (Fig. 3, right column), power to detect the expected 1.85-point difference in ADAS-cog change was 0.80 for the linear mixed effects with categorical time analysis approach and approached 0.80 for the linear mixed effects with continuous time (0.75) and Student’s *t* test (0.70) analysis approaches.

To summarize, the power to detect the expected 1.85-point difference in ADAS-cog change in the planned sample size of 280 is compared across trial-modification scenarios and statistical analysis approaches in Table 3. Acceptable power (i.e., ≥ 0.80) cannot be maintained if the trial is abruptly stopped and no additional outcome data are collected (scenario 1), regardless of the analysis approach taken. Power remains below 0.80 for all analysis methods if there is a 6-month pause in medication and data collection (scenario 2); although, if the analysis approach is changed to a linear mixed effects model with categorical time, power exceeds 0.70. Power also remains below 0.80 for all analysis methods if there is a 6-month pause in medication, but remote assessments are continued on schedule (scenario 3). If the analysis approach is changed to a linear mixed effects model with categorical time, or a linear mixed effects model with continuous time, power exceeds 0.70. The difference in power achieved with scenario 2 and scenario 3 trial modifications is small for either linear mixed effects model with categorical time (.71 vs. .75) or a linear mixed effects model with continuous time (.67 vs. .71) analysis approaches. Acceptable power (i.e., ≥ 0.80) can be obtained in scenarios 2 and 3 by increasing the assumed effect size to a 2.5-point difference in ADAS-cog change or by increasing the sample size (at least for the linear mixed effects model with categorical time analysis approach). Detailed power performance for all three methods at each effect size under sample size of 280 can be found in Table 4.

**Table 3:** Trial construct 2—disease modification trial. Power to detect an expected 1.85-point difference in ADAS-cog change in the planned sample (*n* = 280)

Trial modification	Linear mixed effects model: categorical time	Linear mixed effects model: continuous time	Student’s *t* test	Original *t* test (for reference)
**Scenario 1**	.42	.40	.19	.82
**Scenario 2**	.71	.67	.56	.82
**Scenario 3**	.75	.71	.64	.82

**Table 4:** Trial construct 2, *N* = 280 power performance by methods and scenarios with delta = 1.5, 1.85, 2.0, 2.5

*N* = 280	Effect size	lme cat	lme slp	*t* test	orig
Scenario 0	*D* = 1.5	.77	.75	.66	.66
	*D* = 1.85	.90	.89	.82	.82
	*D* = 2.0	.94	.92	.87	.87
	*D* = 2.5	.99	.99	.97	.97
Scenario 1	*D* = 1.5	.32	.30	.15	.66
	*D* = 1.85	.42	.40	.19	.82
	*D* = 2.0	.46	.44	.22	.87
	*D* = 2.5	.60	.62	.32	.97
Scenario 2	*D* = 1.5	.56	.51	.41	.66
	*D* = 1.85	.71	.67	.56	.82
	*D* = 2.0	.78	.74	.65	.87
	*D* = 2.5	.92	.89	.82	.97
Scenario 3	*D* = 1.5	.59	.55	.46	.66
	*D* = 1.85	.75	.71	.64	.82
	*D* = 2.0	.81	.78	.72	.87
	*D* = 2.5	.94	.92	.88	.97

## Discussion

The COVID-19 pandemic caused unplanned, severe disruptions to the conduct of clinical trials for AD. Our simulations of potential modifications to symptomatic and disease-modifying trials using ADCS legacy trial data enabled us to address a number of fundamental questions that arose from these disruptions. These include (1) whether it would be better to truncate a trial or continue it to a fixed endpoint, perhaps extending the final visit window; (2) would there be sufficient power to detect group differences in longitudinal change with smaller than planned sample sizes and how beneficial it would be to increase the sample size; (3) is there a benefit to incorporating remote outcome assessments during the period of interruption; and (4) what increase in acceptable effect size would be needed if the trial had fewer participants and outcome assessments than anticipated. We were also able to examine the effects of applying potentially more sensitive statistical modeling procedures than simple *t* test comparisons of baseline-endpoint difference scores, such as mixed models of repeated measure (MMRM) treating time as a categorical or continuous variable (i.e., slope).

The results of our simulations of various COVID-related modifications to a 12-month symptomatic treatment trial show that acceptable power (i.e., ≥ 0.80) to detect an expected 2.6-point difference in ADAS-cog change with a planned sample of 360 MCI or early AD patients cannot be maintained if the trial is abruptly stopped and no additional outcome data are collected, regardless of the analysis approach taken. However, acceptable power can be maintained with a 6-month pause in data collection if the analysis approach is changed to a linear mixed effects model with categorical time, and well above acceptable power (i.e., ≥ 0.90) can be maintained with a 6-month pause in data collection, if data collection is resumed after the pause, the trial endpoint is extended, and a linear mixed effects model with categorical time analysis approach is applied. These adaptations would need to be updated in the statistical analytic plan prior to unblinding of the trial. The recent CONSERVE statement provides some of the framework for the trial reporting [4].

Similarly, our results showed that acceptable power (i.e., ≥ 0.80) cannot be maintained if an 18-month AD disease modifying treatment trial with an expected 1.85-point difference in ADAS-cog change and a planned sample of 280 MCI patients is abruptly stopped and no additional outcome data are collected, regardless of the analysis approach taken. If there is a 6-month pause in medication and data collection, power remains below 0.80 for all analysis methods, but increases to just over 0.70 if a linear mixed effects model with categorical time analysis approach is adopted. If outcome assessments are continued remotely during the 6-month medication pause, power remains below 0.80 for all analysis methods, but exceeds 0.70 with linear mixed effects models with categorical or continuous time.

Our results provide information about the relative value of various strategies for mitigating the impact of trial disruption due to COVID-19. First, acceptable power (i.e., ≥ 0.80) can be obtained in an 18-month AD disease-modifying treatment trial with a 6-month pause in medication by increasing the sample size (at least for the linear mixed effects model with categorical time analysis approach). Adding patients to an interrupted trial is usually not a viable option as there most likely has been a considerable time gap between the original completion of randomization and restarting recruitment, resulting in potential selection bias, particularly around a pandemic.

Second, acceptable power (i.e., ≥ 0.80) can be obtained in an 18-month Alzheimer disease-modifying treatment trial with a 6-month pause in medication if the effect size is increased from a 2.0-point to a 2.5-point ADAS-cog difference. If a conservative estimate of expected effect size was used in planning the trial, it may be feasible to increase the expected effect size. Estimation of an expected effect size is often based on results from previous treatment trials (including earlier phase dose finding trials) or determination of a “clinically meaningful” difference on a particular outcome measure.

Third, the difference in power achieved with or without continuing assessments remotely during a 6-month medication pause of our disease-modifying trial scenario is small. The results are similar, regardless of whether a linear mixed effects model with categorical (0.71 vs. 0.75) or continuous time (0.67 vs. 0.71) analysis approach is applied.

The COVID-19 pandemic brought to the fore the need for cognitive outcome measures that are validated for remote assessment. While there is mounting evidence that assessments conducted remotely can yield comparable results to face-to-face assessments in older adults [5] and in patients with MCI and mild AD [6], most of this evidence comes from studies that assess participants via videoconference in a highly controlled and structured in-clinic setting, with the examiner located remotely. Two small validity studies comparing performance on the ADAS-Cog administered in-person and via (in-clinic) videoconference suggest that reliability is high in mildly impaired patients (MMSE > 20) but decreases substantially with increasing dementia severity [5, 6]. There have been few validity studies of remote assessments done with participants located in their own homes and using their own devices. Remote assessment may change the expected psychometric properties of a cognitive outcome measure, changing the mean and variance from those used in designing and powering the trial, leading a bias of effect, inflation of type I error, or a loss of power. Previous work supports the feasibility and utility of home-based cognitive assessments in nondemented individuals [7, 8], but until properly conducted validity studies are completed, it is premature to consider face-to-face and remote, home-based cognitive assessments as comparable for purposes of efficacy analyses.

The choice of statistical analysis approach to be applied is seen in these data to influence the power available to detect a treatment effect in a disrupted trial. In the symptomatic trial, a categorical model appeared to have better power than a slope analysis across the scenarios. By comparison, for the disease-modifying trial, categorical and slope analyses were very similar. The unmodeled *t* test analysis performed worst in both trials and across all scenarios, as expected.

We simulated slightly different expected dropout patterns in the symptomatic and disease-modifying trial constructs. Our sense was that the COVID-19 induced disruption would have a greater impact on more burdensome disease-modifying trials that often requires in-clinic infusion or intravenous administration of medication compared to oral medication generally used in symptomatic trials. With the disease modification trials, we planned dropouts based on the scenario assumptions in the table. Typically, however, dropouts tend to occur early; here, there may have been additional dropouts induced later than usual because of the pandemic. Extending the endpoint of a trial may further dropouts, exacerbating this potential problem.

### Limitations

A strength of the study is that the symptomatic and disease-modifying trial designs were derived from actual trials and for purposes of modeling each design was modified and adjusted to be deidentified so that it could be viewed more broadly.

There are several limitations to this study. First, the datasets used for our simulations were from trials completed over a decade ago, and there may be changes in diagnostic criteria, patient characteristics, and outcome assessment procedures that could reduce the applicability of our results to the current treatment trials. However, we carefully chose ADCS clinical trial datasets for our simulations that used long-standing, standardized assessment procedures and in which participants were demographically and clinically very similar to current early-stage trials.

Second, we did not consider the effect of a treatment/medication hiatus on the outcome of a disrupted treatment trial. It may be the case that under some scenarios, treatment/medication would have to be stopped during a pause while outcome assessments could continue (e.g., our scenario 2 for trial construct 2). Under other scenarios, treatment/medication could continue during a pause, but outcome assessments would have to be stopped. This may be of concern only for treatments that have a cumulative effect over time (e.g., an exercise intervention or monoclonal antibody used to decrease amyloid plaques). Finally, we did not examine the effect of a 12-week visit window extension with a categorical endpoint analysis in the disease-modifying construct.

## Conclusions and implications

The results of our simulations demonstrate the potential utility of various adaptations that might be made to maintain the validity and integrity of treatment trials disrupted by COVID-19 [9], including the use of remote outcome measures and interventions [10]. Clearly, continuing a trial after a pause is a substantially better option than prematurely ending it and analyzing truncated trial data, even in the face of increased dropouts, medication disruptions, missing outcomes, and other exigencies. When there are missing endpoint outcome assessments, adopting a repeated measures statistical analysis approach with either a categorical or continuous outcome is better than applying a simple *t* test approach to examining group differences in baseline-endpoint cognitive outcome measure (e.g., ADAS-Cog) change scores. Extending the duration of the trial to allow more participants to reach endpoint while applying a repeated measures categorical or continuous time statistical analysis approach can be beneficial, but it must be kept in mind that as outcomes are delayed, later assessments have a greater influence on the results of the trial and drop-outs may increase. While many of these modifications may work to maintain power to detect a treatment effect in symptomatic trials, it is less clear that they are effective for disease-modifying trials where expected effect sizes are often smaller, participants are milder, and drop-outs more likely. Adopting remote assessment of a cognitive outcome measure appears to be of limited value in maintaining statistical power. The value of remote assessment may be greater for other common outcome measures (e.g., Clinical Dementia Rating (CDR)), but this awaits further research.

## Data Availability

Databases for the trials are available upon request at https://www.adcs.org/data-sharing/.
